# SLEEP MEDICATION USE AND FALLS IN OLDER ADULTS WITH OSTEOPOROSIS

**DOI:** 10.1093/geroni/igad104.2078

**Published:** 2023-12-21

**Authors:** Loretta Anderson, Stina Ek, Denise Orwig, Alexandra Wennberg

**Affiliations:** University of Maryland Baltimore, Baltimore, Maryland, United States; Karolinska Institutet, Solna, Stockholms Lan, Sweden; University of Maryland School of Medicine, Baltimore, Maryland, United States; Karolinska Institutet, Stockholm, Stockholms Lan, Sweden

## Abstract

One third of older adults take sleep medications, but they are associated with fall risk. One in four older adults experiences a fall each year, costing the U.S. $50 billion dollars annually. Osteoporosis is linked to falls, but little research has examined sleep medication use and falls risk among older adults with osteoporosis. We examined the 2011 National Health and Aging Trends Study (NHATS). Outcomes were falls in the last month, falls in the last year, multiple falls in the last year, and fear of falling. Self-reported sleep medication use dichotomized for analysis (0= once/week or less; 1= 2 or more times/week) was the exposure of interest. Osteoporosis was self-reported (yes/no). Of 8,245 participants, 58% female, 68% white, and 20.5% had osteoporosis. Multivariable logistic regression showed, sleep medication users, compared to rare- or non-users, had higher odds of a fall in the last month (O.R. = 1.64, 95% CI: 1.10, 2.47), in the last 12 months (O.R. = 1.60, 95% CI: 1.20, 2.12), multiple falls in the last 12 months (O.R. = 1.64, 95% CI: 1.08, 2.50), and fear of falling (O.R. = 1.58, 95% CI: 1.18, 2.11). Osteoporotic sleep medication users show increased risk of falls, multiple falls per year, and fear of falling. As older adults with osteoporosis are at increased risk, reducing sleep medication use may decrease this risk, thus decreasing the need for costly medical care. This study contributes to clinicians’ efforts to reduce falls in vulnerable older adults and may reduce overall burden to Medicare.

